# Googling for a veterinary diagnosis: A replication study using Google as a diagnostic aid

**DOI:** 10.1111/jvim.16484

**Published:** 2022-07-11

**Authors:** E. Carley Allen, Kristine M. Alpi, George W. Schaaf, Steven L. Marks

**Affiliations:** ^1^ Red Bank Veterinary Hospitals Tinton Falls New Jersey USA; ^2^ College of Veterinary Medicine North Carolina State University Raleigh North Carolina USA; ^3^ Oregon Health & Science University Portland Oregon USA; ^4^ Wake Forest University School of Medicine Winston Salem North Carolina USA

**Keywords:** differential diagnoses, information resources, open access, problem lists

## Abstract

**Background:**

The purpose of this study was to replicate in the veterinary context a BMJ study using Google to assist in diagnosis of complex cases.

**Hypothesis/Objectives:**

To assess percentage of diagnoses identified using Google as a diagnostic aid in veterinary medicine.

**Animals:**

None; 13 cases in cats and 17 in dogs published in JAVMA.

**Methods:**

Cross‐sectional survey of Google results from searches using keywords generated independently by a generalist and a specialist veterinarian who reviewed the published case history and diagnostic components while blind to the diagnosis. They offered diagnoses and generated up to 5 search strategies for each case. The top 30 Google results for each search were reviewed by the generalist to inform a final Google‐aided diagnosis. Both veterinarians' initial diagnoses and the Google‐aided diagnoses were compared with the published diagnoses.

**Results:**

Google searching led to 52 diagnoses out of 60 possible. Twenty‐two (42%, 95% confidence interval [95% CI] 29%‐55%) Google‐aided diagnoses matched the JAVMA diagnosis. This accuracy rate does not differ significantly from 58% (n = 15/26, 95% CI 38%‐77%) identified in the BMJ study. Google‐aided results were not statistically different from those achieved unaided by each veterinarian (33%, 95% CI 16%‐50%).

**Conclusions and Clinical Importance:**

Published information found searching Google using keywords related to complicated or unusual cases could assist veterinarians to reinforce their initial diagnosis or consider other differential diagnoses. Search strategies using words representing either signs or the preliminary diagnoses can yield results useful to confirming a correct diagnosis.

**Abbreviation**
OA: open access

## 1 INTRODUCTION

Google is used regularly in veterinary medicine by clients and practitioners and in human medicine by patients and practitioners to find medical information. In 2006, the *BMJ* published a research report on the use of Google to help identify complex diagnoses based on cases in the *New England Journal of Medicine (NEJM)*.^1^ The study sample was 26 *NEJM* case record reports and the authors were 2 hospital‐based physicians in the United Kingdom‐a respiratory and sleep physician and a consultant rheumatologist. They found that Google searches yielded the correct diagnoses in 15 of 25 (58%, 95% confidence interval [95% CI] 38%‐77%) cases and suggested that web‐based searching might help doctors to diagnose difficult cases. That study generated many letters and editorials,^2^ and as of June 6, 2022 had 496 citations in Google Scholar. It also inspired similar studies in other medical specialties^3^, ^4^, ^5^, ^6^ and with young physicians and nonphysicians. In veterinary medicine, there are regular discussions on how practitioners can attend to “Dr. Google”^7^ when searched by their clients attempting to diagnose their animals' health conditions, but little discussion of practitioner Google searching behavior in the face of complex cases.

The purpose of this study was to attempt to reproduce the Google Diagnosis study in veterinary medicine to see how the success rates for human medical diagnosis studies compared with diagnosis in veterinary medicine while clarifying some of the methodological limitations of the initial study. While there are many forums for clinicians to discuss differential diagnosis of complex cases, for the purpose of replication, we needed to identify a widely‐disseminated published forum for a general audience of practitioners where challenging cases are presented in veterinary medicine to provide a parallel to the *NEJM* case reports. Many questions about the methodology of the BMJ study arose in our review and replication plan. Ultimately, we wanted to address some of the challenges with the possible impact of clinician expertise on the searches and the final diagnoses.

The overarching research question asked how well Google performs at retrieving content with the correct diagnosis using search terms selected by veterinarians examining a paper case. In addition to the rate of success in retrieving the diagnosis, we wanted to better understand the process and the impact of the search terms chosen and the experience level of the veterinarian using the search results. We hypothesized that (1) search term selection from a case record differs with increased specialization and expertise and (2) that term selection is associated with discoverability of the correct diagnosis. We also wanted to understand whether searching Google provides information that assists in refining initial diagnoses by a generalist or a specialist. If Google succeeds primarily with diagnoses that could already be made confidently without Google, then there is less potential value as a diagnostic tool. We also wanted to learn about the resources retrieved in Google searches that were useful to the diagnostic process.

## 2 MATERIALS AND METHODS

The *New England Journal of Medicine* includes only human diagnostic cases, so for this replication study we reviewed all cases in the What's Your Diagnosis component of the *Journal of the American Veterinary Medical Association* from 2008‐2013 and included 30 cases with dogs or cats being diagnosed for a presumed internal medicine problem. We characterized the cases included in the study (Supplemental data Table 1) by species and age. There were 17 canine and 13 feline cases. The age breakdown was 5 juvenile (under 1 year), 17 adult (aged 1‐12 years), and 8 geriatric (greater than 12 years). Cases included per publication year ranged from 3 in 2009 to 7 in 2013. The diagnoses were then redacted from the cases for presentation to the veterinarians.

A veterinary generalist with 3 years of experience and an internist with 26 years of experience independently examined the redacted cases. For each case, the veterinarians indicated whether the case was familiar to them, gave their top 3 differential diagnoses, and their confidence in their top diagnosis. Parallel to the BMJ study in which the authors selected 3 to 5 search terms from each case record, the veterinarians were asked to provide up to 5 concepts that they would search in Google to look for a diagnosis for the case. The actual instructions for the creation of search terms were: “What terms (up to 5) would you search online to find the diagnosis or differentials for this case? Terms can be phrases or single words; please use 1 line for each term. If you would search a term as a phrase, please put the term in quotes for the purpose of this question (eg, ‘firm subcutaneous mass’).” Two authors quantitatively characterized the search approaches used by the generalist and the specialist looking at concept count, actual word count, inclusion of species (use of common or technical terminology—cat vs feline, dog vs canine) or breed, and inclusion of other signalment information such as age or sex. In counting total words, we counted acronyms or hyphenated words as 1 word, and we included words like “in” and “of” in the count.

The generalist and specialist search terms were searched sequentially in Google on March 10, 2015 on a library‐provided computer between 3:00 pm and 3:55 pm by a single searcher and the first 30 link titles for each search were downloaded as web pages. These search results and the redacted case were provided to the generalist veterinarian for use in assessing whether they contained relevant diagnoses. The generalist veterinarian reviewed all results and marked those they felt contained a relevant diagnosis for up to 3 differential diagnoses. These selections from the results were then compared with the top 3 differential diagnosis from the generalist and specialist based on the case review alone. Two of the authors independently identified the diagnosis from the published cases to establish a definitive diagnosis to serve as the basis of comparison with the diagnoses to be identified from the Google results. For the 10 diagnoses where we were not certain if the generalist‐provided diagnosis was a match with the diagnosis in the article, those cases were adjudicated by the veterinary specialist after the completion of the other study activities. Confidence intervals were calculated and statistical testing of comparisons were made using Z‐tests to compare 2 sample proportions and 2‐sample t‐tests on summary data (means) were performed in Excel or EpiTools epidemiological calculators.^8^

The veterinarians indicated which resource in the Google results they used to provide information for the diagnosis. The librarian and the veterinary student independently categorized those resources by source and level of openness, resolving discrepancies by consensus.

## 3 RESULTS

### 3.1 Initial diagnostic performance

Familiarity with the published cases varied by the veterinary examiner. None of the cases were familiar to the generalist while 10 (33%) were familiar (4 definitely, 6 unsure) to the specialist. The initial diagnoses provided by each veterinarian agreed with the published diagnosis in 10 of 30 cases (33%, 95% CI 16%‐50%). Only 6 cases were correctly diagnosed by both initially. When considering incorrect and correct initial diagnoses, they agreed with each other on 11 cases (37%), and an additional 4 cases had a broader variation of the same diagnosis (e.g., congenital sternal malformation rather than pectus carinatum). Confidence overall about the initial differential diagnoses was similar; the specialist was slightly more confident (33% very confident; 57% somewhat, and 10% not at all) than the generalist (20% very confident, 63% somewhat confident, and 17% not at all confident). Confidence varied by case; they only had the same level of confidence on 16 (53%) of the cases.

### 3.2 Searching strategies

The 2 veterinarians independently generated 102 sets of search terms to be searched in Google. Acronyms such as FIP and TCC were spelled out to “feline infectious peritonitis” and “transitional cell carcinoma.” There was 1 instance where a search strategy returned zero results. Table 1 shows the characteristics of the search approaches used by the generalist and the specialist looking at word count, concept count, inclusion of species (common terminology or adjective—cat vs feline, dog vs canine), inclusion of other signalment factors: age, breed, other, and the number of results retrieved by each set of search terms. On average, the veterinarians used 2.7 concepts ± 1.2 of the allowable 5 concepts compared to 3.2 concepts ± 1.1 in the *BMJ* study. The veterinary searches used more words, 6.6 ± 4.5 compared to the *BMJ* study's 5.0 ± 1.4 but once the veterinary word counts were adjusted to remove the species term included in 78% of searches, the resulting mean 5.8 ± 4.6 words was not different (*P* = .38) The use of age terms and acronyms likely reflected differences in the diseases covered in the human cases and the veterinary cases.

**TABLE 1:** Characteristics of search strategies (N = 102) used in Google by veterinarians seeking information to support their diagnosis of complex cases

Veterinarian	Concepts mean ± SD	Words mean ± SD	Acronyms included	Age term included	Sex term included	Species term included	Technical term use over common term (eg, feline vs cat)	Breed term included
Generalist	4.2 ± 0.9	12.4 ± 4.4	0%	10%	3%	57%	100%	13%
Specialist	2.1 ± 0.5	4.2 ± 1.2	6%	0%	0%	81%	7%	6%

The characterization of the searches by the recent graduate generalist and the specialist yielded significantly different search strategies according to a 2‐sample independent *t* test with unequal variance. The generalist produced 30 searches with a mean of 4.2 (±0.9) concepts compared to the specialist's 72 searches with a mean of 2.1 (±0.5) concepts (*t* = 12.37, *P* < .00001). The average generalist search had 12.4 (±4.3) words (range, 5‐22) compared to the average specialist search with mean 4.2 (±1.2 words) *t* = 9.97, *P* < .00001. The generalist included an age term in 10% and a sex term in 3% of searches while the specialist never included age or sex in the search. Species or breed was mentioned frequently, 63% for the generalist and 81% for the specialist, however the generalist always used feline or canine as the search term while the specialist used the less technical terms cat or dog 93% of the time. However, none of these search characteristics were associated with the percentage of correct Google diagnoses in either the *BMJ* study or our study. The mean number of concepts for incorrect vs correct, was 4.3 vs 4.0 for the generalist and 2.1 vs 1.9 for the specialist. Mean words for incorrect vs correct were 12.3 vs 11.6 for the generalist; the specialist used 4.2 words for both incorrect and correct. From the supplemental data table accompanying the *BMJ* article, we calculated that the human medicine study Google searches used a mean of 5.0 words with a SD of 1.4 words and a range from 2 to 8. Acronyms were used in 15% of searches, and in 2 searches (8%) an age‐related term was added.

### 3.3 Google‐informed diagnostic performance

Supplemental data Table 2 shows the diagnoses and levels of agreement between the unaided diagnoses, the Google‐aided diagnoses, and the actual diagnoses published in JAVMA. The percentage agreement between the diagnosis reached by the generalist veterinarian after examining the Google results and the actual diagnosis was 37.5% (9 of 24) for the generalist search terms and 44.8% (13 of 29) when using the specialist search terms. Discovery and selection of the correct diagnoses in Google results is not significantly associated with the terms used for searching (*z* = 0.5, *P* = .59).

The generalist revised 3 case diagnoses after reviewing Google results from the generalist search terms, 2 were correct (#4 and #29) and 1 was not (#20). Using the Google results from the specialist search terms, 4 were correctly diagnosed (#22, #26, #29, #30) and 1 was incorrectly revised (#4). Of the 5 unique improved diagnoses, for 3 the generalist was originally not at all confident and an additional 2 were originally somewhat confident. The 2 that were changed to be incorrect were somewhat confident originally. Across both sets of search terms, the generalist veterinarian using Google was able to support 46.7% (14 of 30) of the correct diagnoses for this set of cases. Diagnostic success did not increase significantly from initial diagnosis to Google‐aided diagnosis in our sample of 30 cases (*z* = 1.1, *P* = .29). We also found no significant difference (*P* = .33) when comparing the overall proportion of Google‐informed diagnoses (42.3%) with original diagnoses by the generalist and the specialist (33.3% each).

The veterinary diagnosis discovery rate in Google of 42% (95% CI 29%‐55%) does not differ statistically (2‐sample *z* test, *P* = .18) from the human medicine diagnosis discovery rate of 58% (95% CI 38%‐77%).

### 3.4 Characteristics of resources found in Google

From the 103 sets of search results, the generalist selected a resource from the Google results as relevant to informing the ultimate diagnosis in 52 of 60 diagnoses (30 cases by 2 independent veterinarians). Only for a single case (#3) was the same resource site selected from the generalist and specialist sets of search results. A few sites were selected for 2 of the cases in the data set‐‐from the specialist results, Case #7 and #10 relied on the same journal article and Case #17 and #22 used the same page from the National Canine Cancer Foundation.

These resources can be characterized by their formats: books, journals, course notes, continuing education materials, general web pages, but also by the access to their content and the nature of their authorship. Table 2 provides a breakdown of the resource types by access along with a sample item from that type of resource. Of the 6 resources used to revise to a correct diagnosis, 3 were journal abstracts (50%) and the others were 1 open access book (17%), 1 academic website (17%), and the National Canine Cancer Foundation website (17%). Two resources were used to revise to an incorrect diagnosis, an open access journal article and a website intended for practitioners/industry.

**TABLE 2:** Types of veterinary information resources identified from search results as informing generalist's Google‐aided diagnoses, N = 52

Category	Number (%)	Example
Journal Article (Open Access)	12 (23%)	*Journal of Veterinary Internal Medicine* article
Journal Abstract (PubMed/site)	8 (15%)	*Journal of Small Animal Practice* article abstract
Website, Unspecified Authority	8 (15%)	PetWave
Website, Academic/Society	6 (12%)	American College of Veterinary Surgeons
Website, Practice/Industry	4 (8%)	Veterinary Cancer Center
Website, Veterinarian‐Focused (not covered above)	2 (4%)	WikiVet
Book or Book Chapter (Open Access)	3 (6%)	*Textbook of Small Animal Orthopedics*
Book or Book Chapter (Closed Access, snippet only)	3 (6%)	*Fossum's Small Animal Surgery*
Professional magazine	3 (6%)	*DVM360*
Educational Material (CE/Class Notes)	3 (6%)	D.C. Academy of Veterinary Medicine

## 4 DISCUSSION

Searching Google appears to retrieve relevant veterinary resources that assisted a generalist veterinarian in correctly diagnosing 4 challenging cases. The effect is not always an increase; in 2 cases the information led the veterinarian to revise a correct diagnosis to an incorrect one. While diagnostic success from initial diagnosis to Google‐aided diagnosis did not differ significantly in our small sample of cases, we believe that searching on case features is widely done and should be further investigated as a strategy to support generalist practitioners confronted with complex cases.

We hypothesized that term selection differs with increased specialization and expertise. The authors of the human medicine study did not indicate who had performed the searches, just that they searched a mean of 3.2 concepts per search with a minimum of 2 concepts and a maximum of 5 which was the cap set in their methodology that we replicated in our study. This cap artificially constrained the searching flexibility for the clinicians. No numerical search characteristics were associated with the percentage of correct Google diagnoses in either the *BMJ* study or this study. However, the search results generated from terms searched by the specialist veterinarian led to the generalist correcting earlier diagnoses suggesting that modeling how to search more like a specialist could be a useful strategy to consider.

## 5 LIMITATIONS

Replicating the *BMJ* Google Diagnosis study in veterinary medicine was more challenging than expected due to the lack of specificity in the original article about how the authors generated search terms and selected relevant results. We did not contact the *BMJ* authors for more information about their search process or the resources they discovered in Google that contained the diagnoses. Refinement of the Google algorithms over time make it unlikely that we could capture how the algorithm made use of the search history as we progressed through the search. We tried to reduce this effect by doing all the searches at the same time on a single computer by a relatively naive user who had not done many previous veterinary information searches on that machine.

The passage of 9 years from the original *BMJ* study to our searches for comparable veterinary cases is a limitation. Had this been a comparison in human medicine is it likely that the growth of medical information online would result in a different rate of discovery from the human cases. It is likely, but not a testable assumption, that the depth of online veterinary information in 2014 might be comparable to the depth of human medicine online information available back in 2006. It required 6 years to get 30 internal medicine cases from *JAVMA* compared to a single year to get 26 cases from 2005 from the *New England Journal of Medicine*. In part this is because *JAVMA* also publishes cases in other species and other areas of medicine, so only 68% of diagnostic cases published in that timeframe were relevant to our selections.

The greatest limitation to generalizing these findings on Google‐aided diagnosis to the larger veterinary community arose from our focus on replicating the *BMJ* Google Diagnosis. We chose to only use 2 veterinarians in our study to match theirs which involved only 2 clinicians. We recognize that the use of a single generalist and specialist is not representative and that a larger number of observers would have made our study of veterinary diagnoses more robust.

## 6 RELATIONSHIP BETWEEN EXPERTISE, SEARCHING AND DECIDING ON A DIAGNOSIS

The potential impact of specialization and experience length cannot be distinguished based on the nature of the subject matter experts participating in this study. The specialist had 23 more years of experience than the generalist. Search terms varied between the generalist and the specialist. The generalist with fewer years of experience searched items from her problem list, while the search terms presented by the experienced specialist were primarily differential diagnoses themselves. Searching using either signs or differential diagnoses can lead to useful information, but the results can also lead veterinarians to reconsider initial diagnoses that were accurate. Even if Google retrieves information that supports the initial diagnosis, if the diagnosis was already made confidently without Google, then there is potentially little added value for the time spent finding the information. A study of pharmacy students comparing Google with other sources of drug information found that Google did not save time or lead to correct answers for drug questions more than other sources.^9^

Veterinary information resources discoverable by searching Google can assist veterinarians to either reinforce initial diagnosis or consider other diagnoses for complicated or unusual cases.

The lower rate of Google‐aided diagnoses in veterinary medicine compared to human medicine, and the fact that 8 of the searches failed to retrieve helpful information to aid in the diagnosis, suggests that online veterinary information sources have room to grow in their depth and utility.

## CONFLICT OF INTEREST DECLARATION

Authors declare no conflict of interest.

## OFF‐LABEL ANTIMICROBIAL DECLARATION

Authors declare no off‐label use of antimicrobials.

## INSTITUTIONAL ANIMAL CARE AND USE COMMITTEE (IACUC) OR OTHER APPROVAL DECLARATION

Authors declare no IACUC or other approval was needed.

## HUMAN ETHICS APPROVAL DECLARATION

Authors declare human ethics approval was not needed for this study.

## Supporting information


**Supplemental Table 1** Citations, synopses, and characterization by species and age of *JAVMA* What's Your Diagnosis cases selected for this study, N = 30

**Supplemental Table 2** Generalist and specialist initial unaided diagnoses, generalist's Google‐aided diagnoses using generalist or specialist search terms, and agreement with diagnoses published in *JAVMA*

## References

[1] Tang H , Ng JHK . Googling for a diagnosis—use of Google as a diagnostic aid: internet based study. BMJ. 2006;333(7579):1143‐1145. 17098763 10.1136/bmj.39003.640567.AE PMC1676146

[2] Twisselmann B . Use of Google as a diagnostic aid: summary of other responses. BMJ. 2006;333(7581):1270‐1271. 10.1136/bmj.39059.575556.FA PMC1702426 17170420

[3] Falagas ME , Ntziora F , Makris GC , Malietzis GA , Rafailidis PI . Do PubMed and Google searches help medical students and young doctors reach the correct diagnosis? A pilot study. Eur J Intern Med. 2009;20(8):788‐790. 19892310 10.1016/j.ejim.2009.07.014

[4] Jhaveri KD , Schrier PB , Mattana J . Paging doctor Google! Heuristics vs. technology. F1000 Research. 2013;2:90. doi:10.12688/f1000research.2-90.v2 24627777 PMC3907161

[5] Lombardi C , Griffiths E , McLeod B , Caviglia A , Penagos M . Search engine as a diagnostic tool in difficult immunological and allergologic cases: is Google useful? Intern Med J. 2009;39(7):459‐464. 19664156 10.1111/j.1445-5994.2008.01875.x

[6] Siempos II , Spanos A , Issaris EA , Rafailidis PI , Falagas ME . Non‐physicians may reach correct diagnoses by using Google: a pilot study. Swiss Med Wkly. 2008;138(49–50):741‐745. 19130327 10.4414/smw.2008.12320

[7] Niedziela K. British vets forced to compete with ‘Dr. Google.’ *Vet Pract News* . https://www.veterinarypracticenews.com/british-vets-forced-to-compete-with-dr-google/. Accessed August 18, 2014.

[8] Sergeant ESG . Epitools Epidemiological Calculators . AusVet Animal Health Services and Australian Biosecurity Cooperative Research Centre for Emerging Infectious Disease; 2015. http://epitools.ausvet.com.au. Accessed June 28, 2022.

[9] Giuliano C , McConachie S , Kalabalik‐Hoganson J . Multicenter randomized comparative trial of Micromedex, Micromedex with Watson, or Google to answer drug information questions. J Med Libr Assoc. 2021;109(2):212‐218. doi:10.5195/jmla.2021.1085 34285664 PMC8270367

