# Supplementary material for: Googling for a veterinary diagnosis: A replication study using Google as a diagnostic aid
Source: J Vet Intern Med. 2022 Jul 11;36(4):1466–70. doi: 10.1111/jvim.16484 (PMC9308411; doi:10.1111/jvim.16484)
Supplement: Supplementary file 1 — Supplemental Table 1 Citations, synopses, and characterization by species and age of JAVMA What's Your Diagnosis cases selected for this study, N = 30 [file JVIM-36-1466-s001.pdf]

Supplemental Data Table 1. Citations, synopses, and characterization by species and age of *JAVMA* What's Your Diagnosis cases selected for this study, N=30

| Case ID | Authors | Year | DOI | Case Synopsis | Species | Age |
|---|---|---|---|---|---|---|
| 1 | Frankel CS, Young TL, Alvarez-Berger F, Spencer CP | 2013 | 10.2460/javma.243.3.329 | castrated male DSH feline with right hind lameness, difficulty jumping, decreased appetite and constipation | Feline | Adult |
| 2 | Nemanic S, Nelson NC, Guiot LP | 2013 | 10.2460/javma.243.5.627 | spayed female DLH feline with weight loss, tenesmus, elevated ALT and elevated ALKP | Feline | Geriatric |
| 3 | Monibi FA, Tromblee TC | 2013 | 10.2460/javma.242.2.157 | spayed female Bichon Frise with abdominal pain, lethargy, inappetence and vomiting | Canine | Adult |
| 4 | Agut A, Boza S, Murciano J, Belda E, Soler M | 2013 | 10.2460/javma.242.6.745 | intact male German Shepherd Dog with right hind lameness | Canine | Adult |
| 5 | Harran N, Bowlt K, Llabres-Diaz F, Daly M, Barr F | 2013 | 10.2460/javma.242.7.917 | spayed female Siamese with swelling of the left zygomatic arch and left forelimb lameness | Feline | Adult |
| 6 | Hanson J, Latouche JS, Chamberlin N | 2013 | 10.2460/javma.242.12.1637 | spayed female DSH with decreased appetite, lethargy and polydipsia | Feline | Adult |
| 7 | Williamson BG, O'Brien DP | 2013 | 10.2460/javma.242.12.1641 | castrated male DSH with hypnic jerks | Feline | Juvenile |
| 8 | Aulakh HK, Tyson R, Aulakh KS, Archipow W | 2012 | 10.2460/javma.241.3.319 | male German Shepherd Dog with acute vomiting | Canine | Adult |
| 9 | Thomas RM, Fischetti AJ. | 2012 | 10.2460/javma.241.7.881 | spayed female DSH with stranguria, tenesmus, vomiting and open-mouthed breathing | Feline | Geriatric |
| 10 | Berthiaume DR, Kline KL | 2012 | 10.2460/javma.241.11.1437 | spayed female DMH with stupor, blindness, and recent onset of seizure-like activity | Feline | Adult |

| | | | | | | |
|---|---|---|---|---|---|---|
| 11 | Haynes KH, Cavanaugh RP, Steinheimer D | 2012 | 10.2460/javma.241.11.1433 | female spayed DSH with a painful hypodermal mass of the distal left antebrachium | Feline | Geriatric |
| 12 | Arnold EJ, Pressler BM, Heng HG | 2012 | 10.2460/javma.240.7.821 | spayed female Miniature Schnauzer with vomiting, lethargy and bilateral organomegaly | Canine | Adult |
| 13 | Delisser PJ, Burton NJ | 2012 | 10.2460/javma.240.11.1289 | castrated male Burmese cat with reluctance to jump and right-sided plantigrade stance | Feline | Adult |
| 14 | Durden AC, Kent M, Platt SR | 2011 | 10.2460/javma.239.3.303 | spayed female Labrador Retriever with exophthalmos and epiphora of the right eye | Canine | Geratric |
| 15 | Schwartz Z, Beale BS | 2011 | 10.2460/javma.238.5.565 | intact male Pug with a protrusion over the caudal sternum | Canine | Juvenile |
| 16 | Aulakh KS, Harper TA, Horowitz FB, Tyson R | 2011 | 10.2460/javma.238.6.699 | castrated male Golden Retriever with 5-day history of regurgitation after eating | Canine | Adult |
| 17 | Cronin KL, Williams J, Klose T | 2011 | 10.2460/javma.238.9.1107 | spayed female German Shepherd Dog with lethargy, inappetence and weight loss | Canine | Adult |
| 18 | Ranade SA, Pacchiana PD | 2011 | 10.2460/javma.238.10.1243 | castrated male DSH with progressive right hind limb lameness | Feline | Geriatric |
| 19 | Almondia DY, Williams J | 2010 | 10.2460/javma.237.2.159 | spayed female mixed-breed dog with transitional cell carcinoma of the bladder and recurrent urinary tract infections | Canine | Adult |
| 20 | Bolduc KM | 2010 | 10.2460/javma.237.7.781 | intact male stray DSH with a 1-week history of coughing | Feline | Adult |
| 21 | Johnson PJ, Hayward NJ | 2010 | 10.2460/javma.236.2.169 | male DLH with vomiting, inappetance and lethargy | Feline | Juvenile |

| | | | | | | |
|---|---|---|---|---|---|---|
| 22 | Leach LM, Van Camp SL | 2010 | 10.2460/javma.236.8.827 | castrated male Labrador Retriever with a nonproductive cough | Canine | Geriatric |
| 23 | Brown EM, Rademacher N, Gieger TL, Gaschen LE, Buchholz J | 2010 | 10.2460/javma.236.9.953 | female DSH with a history of nasal lymphoma and acute respiratory distress | Feline | Adult |
| 24 | Little AM, Hecht S, Kirk CA, Bohling MW | 2009 | 10.2460/javma.235.9.1041 | castrated male Labrador Retriever with weight loss, anorexia, labored breathing, dry productive cough, and pleural effusion | Canine | Geriatric |
| 25 | Downs MO, Houghton JO | 2009 | 10.2460/javma.234.3.327 | spayed female Rhodesian Ridgeback with polyuria and polydipsia | Canine | Adult |
| 26 | Erne JB, McNicholas WT | 2009 | 10.2460/javma.234.2.201 | intact male Yorkshire Terrier with intermittent dysuria | Canine | Adult |
| 27 | Astor DE, Valdes-Martinez A, Hoelzler MG | 2008 | 10.2460/javma.233.12.1849 | intact male Rottweiler with left pelvic limb lameness | Canine | Juvenile |
| 28 | Rebhun RB, Greenberg MJ, Silver TI | 2008 | 10.2460/javma.233.11.1691 | spayed female Labrador Retriever with incomplete resection of an intermediate grade peripheral nerve sheath tumor | Canine | Geriatric |
| 29 | Agut A, Haro P, Soler M, Murciano J, Belda E, Buendía A | 2008 | 10.2460/javma.233.8.1235 | female Golden Retriever dog with deformities of the thorax and limbs. | Canine | Juvenile |
| 30 | Coleman KD, Lurie DM, Blaik MA, Demuth G, Kirkby K | 2008 | 10.2460/javma.232.12.1801 | spayed female Golden Retriever dog with right forelimb lameness, associated muscle atrophy and mass on right shoulder. | Canine | Adult |
