# Supplementary material for: Googling for a veterinary diagnosis: A replication study using Google as a diagnostic aid
Source: J Vet Intern Med. 2022 Jul 11;36(4):1466–70. doi: 10.1111/jvim.16484 (PMC9308411; doi:10.1111/jvim.16484)
Supplement: Supplementary file 2 — Supplemental Table 2 Generalist and specialist initial unaided diagnoses, generalist's Google‐aided diagnoses using generalist or specialist search terms, and agreement with diagnoses published in JAVMA [file JVIM-36-1466-s002.pdf]

Supplemental Data Table 2
Generalist and specialist initial unaided diagnoses, generalist's Google-aided diagnoses using generalist or specialist search terms, and agreement with diagnoses published in JAVMA.

**ID**	Generalist Initial Dx	Specialist Initial Dx	Google-aided Dx using Generalist Terms (GAD-GT)	Google-aided Dx using Specialist Terms (GAD-ST)	JAVMA Diagnosis	GAD-GT	GAD-ST	Either GAD-GT or GAD-ST
						Agreement with JAVMA diagnosis		
**1**	Neoplasia	lymphoma	no	Osteosarcoma	Osteosarcoma	NA	Y	Y
**2**	Metastatic Neoplasia	mast cell disease	lymphosarcoma	Mast Cell Tumor	Myelolipoma	N	N	N
**3**	Left hydronephrosis	obstructive uropathy	hydronephrosis secondary to ureterolithiasis	Hydronephrosis secondary to ureterolithiasis	Hydronephrosis, secondary to a transitional cell carcinoma	N	N	N
**4**	Soft tissue sarcoma	osteosarcoma	fungal (blastomycosis) osteomyelitis	osteosarcoma	Osteomyelitis - Cryptococcosis	Y	N	Y
**5**	Disseminated fungal disease	osteosarcoma	osteosarcoma	osteosarcoma	Tuberculosis - Mycobacterium bovis	N	N	N
**6**	metastatic lymphoma	lymphoma	no	lymphoma	Adenocarcinoma	NA	N	N
**7**	Congenital brain malformation/hydrocephalus	inflammatory brain disease	hydrocephalus	Feline infectious peritonitis	Porencephaly	N	N	N
**8**	GI foreign body/obstruction	GI obstruction	small intestinal obstruction	small intestinal obstruction	Abdominal adhesions	N	N	N

**9**	Metastatic neoplasia (lymphoma, mast cell tumor)	mast cell	no	splenic hemangiosarcoma	Myelolipoma	N	N	N
**10**	Infectious Encephalomyelitis (FIP)	Lymphoma	feline infectious peritonitis	Feline infectious peritonitis	Meningitis - Cryptococcus gattii	N	N	N
**11**	bengin osseous proliferation	osteosarcoma	osteoma	osteoma	Osteoma (benign osseous Tumor)	Y	Y	Y
**12**	metastatic lymphoma	renal carcinoma	metastatic lymphoma	lymphoma	histiocytic sarcoma	N	N	N
**13**	Intervertebral disk herniation at L6-L7	disc disease in cats	intervertebral disk herniation	intervertebral disk disease	Lateralized intervertebral disk extrusion (Hansen type 1)	Y	Y	Y
**14**	Orbital neoplasia	Neoplasia	no	orbital meningioma	cavernous sinus syndrome	NA	N	N
**15**	pectus carinatum	congenital sternal malformation	pectus carinatum	pectus carinatum	pectus carinatum	Y	Y	Y
**16**	hiatal hernia	neoplasia	carcinoma	caudal esophageal neoplasia	*mediastinal cyst*	N	N	N
**17**	thymic neoplasia	lymphoma	lymphoma	lymphoma	Thymic lymphoma	Y	Y	Y
**18**	soft tissue sarcoma	osteosarcoma	no	soft tissue sarcoma	Solitary osteochondroma	NA	N	N
**19**	recurrence of TCC mass compressing colon	TCC	recurrent transitional cell carcinoma	transitional cell carcinoma	Emphysematous cystitis	N	N	N

**20**	paragonamous kellicotti	paragonimus	no	paragonimus	Lung flukes - Paragonimus kellicotti	NA	Y	Y
**21**	linear small intestinal foreign body	linear foreign body	linear small intestinal obstruction	linear foreign body	Linear foreign body	Y	Y	Y
**22**	metastatic neoplasia	pulmonary carcinoma	mycobacterium tuberculosis	lymphoma	Pulmonary lymphomatoid granulomatosis - pulmonary lymphosarcoma	N	Y	Y
**23**	mediastinal lymphoma compressing trachea	lymphoma	esophageal lymphoma	tracheal lymphoma	Tracheal neoplasia - lymphoma	Y	Y	Y
**24**	trauma-induced diaphragmatic hernia	diaphragmatic hernia	diaphragmatic hernia	diaphragmatic hernia	Diaphragmatic hernia	Y	Y	Y
**25**	paraneoplastic hypercalcemia (lymphoma)	thymoma	lymphoma	lymphoma	Thymoma	N	N	N
**26**	urinary tract stones (urethrolith)	urethral mass associated with os penis	urethral neoplasia	urethral obstruction secondary to os penis fracture scar tissue formation	fibrous urethral stricture (obstruction) secondary to trauma (fractured os penis)*	N	Y	Y
**27**	avascular necrosis of the femoral head	metabolic bone disease	no	no	osteomyelitis (Septic arthritis)	NA	NA	N

**28**	metastatic neoplasia (nerve sheath tumor)	nerve sheath tumor metastasis	thymoma	thymoma	Intrathoracic (mediastinal) lipomas	N	N	N
**29**	dwarfism	epiphyseal dysplasia	multiple epiphyseal dysplasia	multiple epiphyseal dysplasia	multiple epiphyseal dysplasia	Y	Y	Y
**30**	peripheral nerve sheath tumor	soft tissue sarcoma	nerve sheath tumor	soft tissue sarcoma	histiocytic sarcoma	N	Y	Y
					NA	6	1	0
					Yes	9	13	14
					No	15	16	16
						37.5%	44.8%	46.7%

NA – Not applicable means a Google-aided diagnosis was not generated. Y = Yes, agreement. N = No, disagreement
